# The potent tumor suppressor miR-497 inhibits cancer phenotypes in nasopharyngeal carcinoma by targeting *ANLN* and *HSPA4L*

**DOI:** 10.18632/oncotarget.5651

**Published:** 2015-10-14

**Authors:** Shumin Wang, Yingxi Mo, Kaoru Midorikawa, Zhe Zhang, Guangwu Huang, Ning Ma, Weilin Zhao, Yusuke Hiraku, Shinji Oikawa, Mariko Murata

**Affiliations:** ^1^ Department of Environmental and Molecular Medicine, Mie University Graduate School of Medicine, Tsu, Mie, Japan; ^2^ Department of Otolaryngology Head and Neck Surgery, First Affiliated Hospital of Guangxi Medical University, Nanning, Guangxi, China; ^3^ Faculty of Nursing Science, Suzuka University of Medical Science, Suzuka, Mie, Japan

**Keywords:** nasopharyngeal carcinoma, microRNA, Epstein-Barr virus, tumor suppressor, biomarker

## Abstract

Nasopharyngeal carcinoma (NPC) is a malignancy with poor prognosis that is endemic to Southeast Asia. We profiled microRNAs (miRNAs) of NPCs using microarrays and confirmed the results by quantitative RT-PCR. The results revealed that seven miRNAs were significantly up-regulated, and six miRNAs were down-regulated, in NPC tissues relative to noncancerous nasopharyngeal epithelia (NNE). Expression of miR-497 was also significantly reduced in the plasma of NPC patients relative to the plasma of noncancerous control patients. The concordant down-regulation of miR-497 in tissues and plasma suggested that miR-497 could be used as a diagnostic biomarker for NPC. Functional analyses of the effect of miR-497 on cancer phenotypes revealed that transfection of miR-497 mimic into NPC cells suppressed cell growth and migration and induced apoptosis. Subcutaneous xenografts of transfected cells in nude mice demonstrated that miR-497 significantly inhibited tumor growth. Two potential targets of miR-497, *ANLN* (anillin, actin-binding protein) and *HSPA4L* (heat shock 70 kDa protein 4–like), both of which were overexpressed in NPC tissues, were negatively regulated by miR-497 mimic in NPC cell lines. Silencing of *ANLN* and *HSPA4L* suppressed cell proliferation and migration and induced apoptosis in NPC cells. Our findings indicate that miR-497 is a potent tumor suppressor that inhibits cancer phenotypes by targeting *ANLN* and *HSPA4L* in NPC.

## INTRODUCTION

MicroRNAs (miRNAs) are non-protein-coding small RNAs, 19–25 nucleotides (nt) in length, that are cleaved from 70–100 nt hairpin pre-miRNA precursors [1]. miRNAs negatively regulate gene expression primarily by binding to the 3′ untranslated regions (3′UTR) of their target messenger RNAs (mRNAs), leading to translational repression or mRNA cleavage [2]. Recent studies revealed that miRNAs regulate the expression of a wide variety of target genes, and are thus involved in a wide range of biological processes including cell proliferation, differentiation, and apoptosis [3–5]. In addition to their physiological functions, the wide-ranging biological effects of miRNAs can be also involved in cancer development [6, 7].

Nasopharyngeal carcinoma (NPC) is a rare disease in most parts of the world [8], but in Southern China and Southeast Asia it is one of the most prevalent malignant tumors and the leading cause of death among all head and neck cancers. Furthermore, this malignancy tends to be diagnosed at an advanced stage, and consequently has a poor prognosis. Currently, there is no reliable biomarker for early detection of NPC, and the underlying molecular mechanisms are poorly understood, making it difficult to develop effective therapeutic strategies. NPC is a complex disease resulting from a multi-step process of carcinogenesis that involves interactions among chronic Epstein-Barr virus (EBV) infection, environmental factors, genetic mutation, and epigenetic changes [9, 10]. Previously, in a study aimed at characterizing the epigenetic changes in NPC, we identified several candidate genes whose promoters are aberrantly methylated during NPC carcinogenesis [11–13]. However, it is necessary to thoroughly elucidate epigenetic mechanisms, including miRNAs, as well as DNA methylation. NPC is typically diagnosed at advanced stages; therefore, it is desirable to identify novel biomarkers including circulating miRNAs as clinical tools, and clarifying the mechanisms of carcinogenesis can facilitate the development of therapeutic targets.

In order to investigate the role of miRNAs in NPC pathogenesis, we performed microRNA profiling and quantitative RT-PCR to evaluate the expression levels of candidate miRNAs in a set of NPC primary tumor biopsies and plasma. The results of these analyses revealed concordant down-regulation of miR-497 in tissues and plasma. Using the TargetScan algorithm, we identified two genes, *ANLN* (anillin, actin-binding protein) and *HSPA4L* (heat shock 70 kDa protein 4–like) as potential targets of miR-497. Quantitative RT-PCR and immunohistochemical analyses confirmed that *ANLN* and *HSPA4L* were overexpressed in NPC tissues relative to NNE tissues. Transfection of a miR-497 mimic or small interfering RNAs (siRNAs) against *ANLN* and *HSPA4L* inhibited cancer phenotypes in NPC cells. Our findings suggest that miR-497 acts as a tumor suppressor by targeting *ANLN* and *HSPA4L* in NPC.

## RESULTS

### Identification of miRNAs differentially expressed in NPC tissues

We performed miRNA microarray analysis of clinical samples of seven NPC patients and five patients without cancer. Thirty-six EBV-related miRNAs were overexpressed in NPC, and we chose the top three miRNAs (Table 1A) as candidates for further studies. In addition, we selected the top four miRNAs among the 11 up-regulated human miRNAs, and the top eight miRNAs among the 66 down-regulated human miRNAs (Table 1B).

**Table 1:** Candidate miRNAs analyzed by miRNA microarray

miRNA name	mirbase accession No.	Fold change of NPC compared with NNE (log2)	*P*-value
**(A) Epstein-Barr virus-related miRNAs**			
ebv-miR-BART22	MIMAT0010132	13.57	1.70E-09
ebv-miR-BART1-3p	MIMAT0003390	13.16	5.29E-09
ebv-miR-BART9	MIMAT0003419	13.12	2.99E-09
**(B) Human miRNAs**			
**1) Up-regulated miRNAs**			
hsa-miR-205	MIMAT0000266	2.57	2.47E-05
hsa-miR-182	MIMAT0000259	1.70	0.00134
hsa-miR-135b	MIMAT0000758	1.46	0.01364
hsa-miR-455-3p	MIMAT0004784	1.26	0.04598
**2) Down-regulated miRNAs**			
hsa-miR-145	MIMAT0000437	−2.81	0.00407
hsa-miR-497	MIMAT0002820	−2.51	0.00107
hsa-miR-150	MIMAT0000451	−2.42	0.01117
hsa-miR-195	MIMAT0000461	−2.27	0.00076
hsa-miR-342-5p	MIMAT0004694	−2.25	0.00939
hsa-miR-143	MIMAT0000435	−2.22	0.00311
hsa-miR-34b*	MIMAT0000685	−2.20	0.04503
hsa-miR-100	MIMAT0000098	−2.10	0.00063

**Figure 1:**
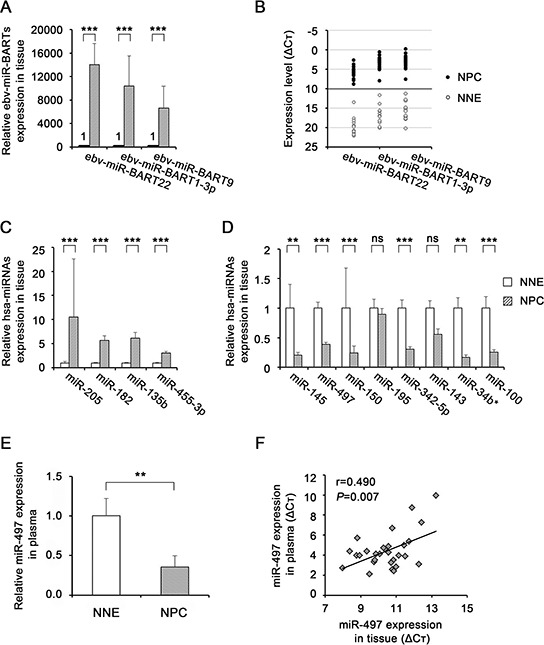
miRNA expression levels in nasopharyngeal tissues and plasma miRNA expression levels of ebv-miR-BARTs **(A, B)** and human miRNAs **(C, D)** were determined by quantitative RT-PCR in NNE tissues (*n* = 11) and primary NPC tissues (*n* = 18) normalized against the corresponding levels of *RNU6B*. The relative quantity in NNE was defined as 1 (A, C, D). **B.** Graph shows ebv-miR-BARTs data in ΔCt scale (open circle: NNE, closed circle: NPC). **E.** miR-497 expression levels were determined by quantitative RT-PCR in plasma of the same patients mentioned above. The levels were normalized using the average of five reference miRNAs, and the relative quantity in NNE was defined as 1. *P*-values of differences between NPC and NNE were calculated using Student's *t*-test (**P < 0.01, ***P < 0.001). **F.** Pearson correlation of miR-497 expression (expressed on the ΔCt scale) between tissues and plasma.

To confirm the candidate dysregulated miRNAs identified by miRNA microarray analysis, we performed quantitative RT-PCR on 18 NPC and 11 NNE tissue samples. All three of the selected ebv-miR-BARTs (BamHI A Rightward Transcripts; ebv-miR-BART22, ebv-miR-BART1-3p, and ebv-miR-BART9) were more highly expressed in NPC tissues than in NNE (Fig. 1A). In addition, ΔCt values clearly distinguished between NPC (always <10) and NNE (always >10) (Fig. 1B). The quantitative RT-PCR analysis confirmed that all four of the selected up-regulated hsa (*Homo sapiens*)-miRNAs (miR-205, miR-182, miR-135b, and miR-455-3p) were significantly up-regulated in NPC tissues (Fig. 1C). We confirmed that six of the eight selected down-regulated hsa-miRNAs (miR-145, miR-497, miR-150, miR-342-5p, miR-34b* and miR-100) were significantly down-regulated in NPC tissues, whereas miR-195 and miR-143 exhibited no significant difference between the two groups of subjects (Fig. 1D).

### Circulating miR-497 is significantly down-regulated in plasma of NPC

We also measured the levels of miRNAs in plasma of NPC patients and noncancerous controls by quantitative RT-PCR. The five reference miRNAs exhibited no significant difference in plasma levels between NNE and NPC (Supplementary Fig. 1). One of the the 13 dysregulated miRNAs detected in tissues, miR-497, was present at significantly lower levels in NPC plasma than in noncancerous control plasma (*P* < 0.01, Fig. 1E), whereas the levels of other miRNAs were not significantly altered (Table S1). Expression of miR-497 was significantly correlated between tissues and plasma (Fig. 1F, Pearson correlation coefficient; *r* = 0.490, *P* = 0.007), whereas the other miRNAs exhibited no significant correlation (Table S1). Thus, miR-497 was concordantly down-regulated in tissues and plasma, suggesting that it would could be useful as a biomarker for NPC. Therefore, we focused on miR-497 in our subsequent exploration of miRNA functions in NPC.

### Exogenous miR-497 suppresses cell proliferation and migration, and induces apoptosis, in NPC cell lines

**Figure 2:**
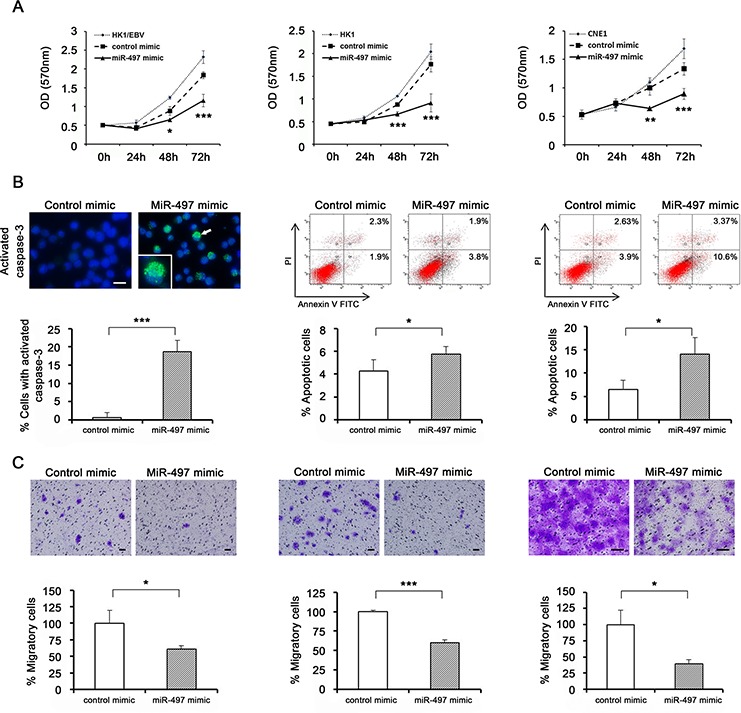
Functional analyses using transfection of exogenous miRNA **A.** Cell proliferation was determined by MTT assay every 24 h for 72 h after transfection of HK1/EBV, HK1, and CNE1 cells with miRNA mimic (*n* = 5). **B.** Apoptotic cells were detected by immunocytochemical staining (left) and flow cytometry (middle and right). miR-497 mimic induced activation of caspase-3 protein, which exhibited cytoplasmic localization (an arrow and the enlarged). HK1/EBV cells were stained with DAPI and photographed under a fluorescence microscope after transfection (*n* = 4). HK1 and CNE1 cells were transfected with control or miR-497 mimic, and then stained with annexin V-FITC and PI and subjected to flow cytometry (*n* = 3). **C.** Cell migration in NPC cells transfected with miR-497 mimic. HK1/EBV (left), HK1 (middle), and CNE1 (right) cells were transfected with control or miR-497 mimic, and then cultured for 72 h. After transfection, harvested cells were seeded in a migration chamber and cultured for 24 h. Migratory cells were stained, photographed under a microscope, and quantitatively analyzed (*n* = 3). Data are shown as means ± standard deviation. *P*-values were calculated using Student's *t*-test (*P < 0.05, ***P < 0.001) compared with control mimic. Scale bar represents 50 μm.

Prior to our *in vitro* functional analyses, we confirmed by quantitative RT-PCR that miR-497 was down-regulated in NPC cell lines, as it is in NPC tissues relative to NNE tissues (Supplementary Fig. 2A). To investigate the biological function of miR-497 down-regulation in NPC, we assessed the effect of exogenously administered miRNA on cell proliferation using the MTT assay. Specifically, we transfected miR-497 mimic and negative control into NPC cell lines, and confirmed the expression level at the indicated time points (Supplementary Fig. 2B-2D). Compared to the negative control, miR-497 mimic significantly inhibited growth of HK1/EBV, HK1, and CNE1 cells (Fig. 2A, left, middle, right, respectively), suggesting that miR-497 has a tumor-suppressive function.

Caspase-3, which is activated in apoptotic cells, was observed in the cytoplasm of HK1/EBV cells transfected with miR-497 mimic, but not in cells transfected with control mimic. Furthermore, the percentage of cells with activated caspase-3 was significantly higher in miR-497 mimic–transfected cells (Fig. 2B, left). The degree of early apoptosis was determined based on the percentage of the annexin V-FITC-positive and PI-negative cells, and late apoptosis was determined based on the percentage of the annexin V-FITC-positive and PI-positive cells. The overall apoptosis rate was the sum of the early- and late-apoptotic subpopulations. In HK1 (Fig. 2B, middle) and CNE1 cells (Fig. 2B, right) transfected with miR-497 mimic, the apoptosis rate was significantly higher than in cells transfected with control mimic, suggesting the involvement of miR-497 in inducing apoptosis in NPC cells.

In addition, we carried out cell migration assays using NPC cell lines 72 h after transfection with miR-497 or control mimic. The assay kit contained a polycarbonate membrane insert (pore size, 8 μm) in each well, which served as a barrier to discriminate migratory from non-migratory cells. Migratory cells were observed less frequently among NPC cells transfected with miR-497 mimic than among those transfected with control mimic (Fig. 2C).

### MiR-497 inhibited tumor growth *in vivo*

**Figure 3:**
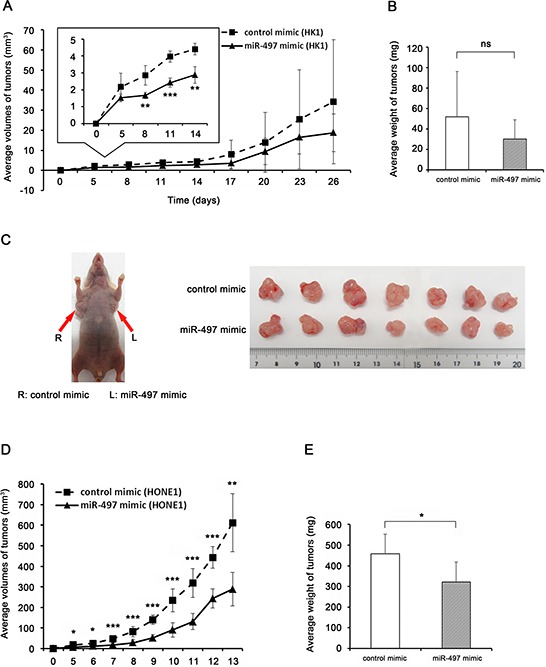
miR-497 inhibits NPC tumor growth *in vivo* **A, D.** Growth curve drawn by measuring tumor volumes at the indicated times (A inset: enlarged view of growth curve). **B, E.** Weight of xenograft tumors. Data are shown as means ± standard deviation (B: *n* = 4, E: *n* = 7). **C.** Image of subcutaneous xenografts in the mouse flanks (the arrows indicate the presence of tumors in mouse injected with control mimic–transfected cells (R) and miR-497 mimic–transfected cells (L)) and excised tumors. *P*-values of differences between miR-497 and control tumors were calculated using the Student's *t*-test (*P < 0.05, **P < 0.01, ***P < 0.001; ns, no significant difference).

To examine the role of miR-497 in NPC development, we performed a xenograft study in which miR-497 mimic– or control mimic–transfected HK1 and HONE1 cells were transplanted into the flanks of BALB/c athymic nu/nu mice. Subcutaneous tumor growth of miR-497 mimic–transfected cells was slower than that of control mimic–transfected cells, and tumor volumes were significantly smaller until day 14 (HK1: Fig. 3A inset, HONE1: Fig. 3D). Because tumor growth of HK1 cells was relatively slow, we continued the observation after day 14, but thereafter there was no significant difference in the volumes of HK1 xenografts (Fig. 3A) or tumor weight at sacrifice (day 26: miR-497 mimic, 30.0 ± 19.0 mg vs. control mimic, 52.0 ± 44.1 mg; *P* = 0.395), as shown in Fig. 3B. By contrast, tumor xenografts of HONE 1 cells grew rapidly, and mice were sacrificed for evaluation of tumor formation 13 days after inoculation (Fig. 3C). The tumor weight of HONE1 xenografts was significantly lower in the miR-497 mimic tumors than in the control mimic tumors (322.8 ± 94.5 mg vs. 457.9 ± 95.4 mg, *P* = 0.02, Fig. 3E). In addition, we confirmed that miR-497 was expressed at significantly higher levels in the miR-497 mimic tumors at day 13 (Supplementary Fig. 2E). The tumor xenograft experiment indicated that miR-497 plays a role in suppressing NPC tumorigenicity.

### Up-regulation of target genes ANLN and HSPA4L in NPC tissues

To search for target genes of miR-497, we used TargetScan Human (Release 6.2: June 2012, http://www.targetscan.org/vert_61/). Of 1273 conserved predicted target genes, 44 were on the list of genes that were up-regulated (>2-fold, *P* < 0.05) in NPC vs NNE tissues, as determined by mRNA microarray experiments. Based on a literature survey, we focused our attention on *ANLN* (anillin, actin-binding protein) and *HSPA4L* (heat shock 70 kDa protein 4–like) because these two genes are up-regulated in many cancers [14–18].

**Figure 4:**
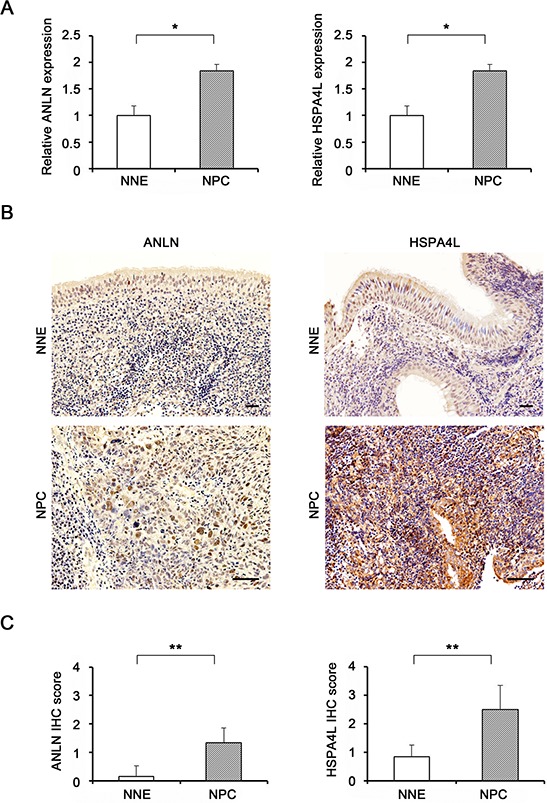
Target gene expression levels in NPC tissues **A.** mRNA expression levels of *ANLN* (left) and *HSPA4L* (right) in NNE tissues (*n* = 11) and primary NPC tissues (*n* = 18), determined by quantitative RT-PCR. Expression levels of target genes (*ANLN* and *HSPA4L*) were normalized against the corresponding levels of *GAPDH*. *P*-values were calculated using Student's *t*-test (*P < 0.05). **B.** Formalin-fixed and paraffin-embedded biopsies of nasopharyngeal tissues were obtained from patients with chronic nasopharyngitis (NNE) or NPC. Expression of ANLN (left) and HSPA4L (right) was assessed by immunoperoxidase staining (brown). Original magnifications are 100× and 200 ×. Scale bar represents 50 μm. **C.** Graphs represent average and SD of IHC scores for NNE (*n* = 6) and NPC tissues (*n* = 6). *P*-values of differences between NPC and NNE tissues were calculated using the Mann–Whitney U test (**P < 0.01).

We measured expression levels of *ANLN* and *HSPA4L* in NPC and NNE tissues using quantitative RT-PCR and protein levels by immunohistochemistry (IHC). As shown in Fig. 4A, mRNA levels of *ANLN* and *HSPA4L* was significantly higher in NPC tissues than in NNE tissues. IHC analyses revealed up-regulation of both proteins in NPC. By contrast, in NNE tissues, little or no immunoreactivity of ANLN was observed (Fig. 4B, left upper), and HSPA4L-expressing cells were scarce (Fig. 4B, right upper). In primary NPC tissues, ANLN was expressed in the cytoplasm of NPC tumor cells (Fig. 4B, left lower), whereas HSPA4L was strongly expressed in the nucleus and cytoplasm of NPC cells and mucosa adjacent to NPC nests (Fig. 4B, right lower). IHC scores of both proteins were significantly higher in NPC than in NNE (*P* = 0.003 and *P* = 0.002, respectively, Fig. 4C).

### Significant down-regulation of ANLN and HSPA4L in NPC cells by exogenous miR-497

**Figure 5:**
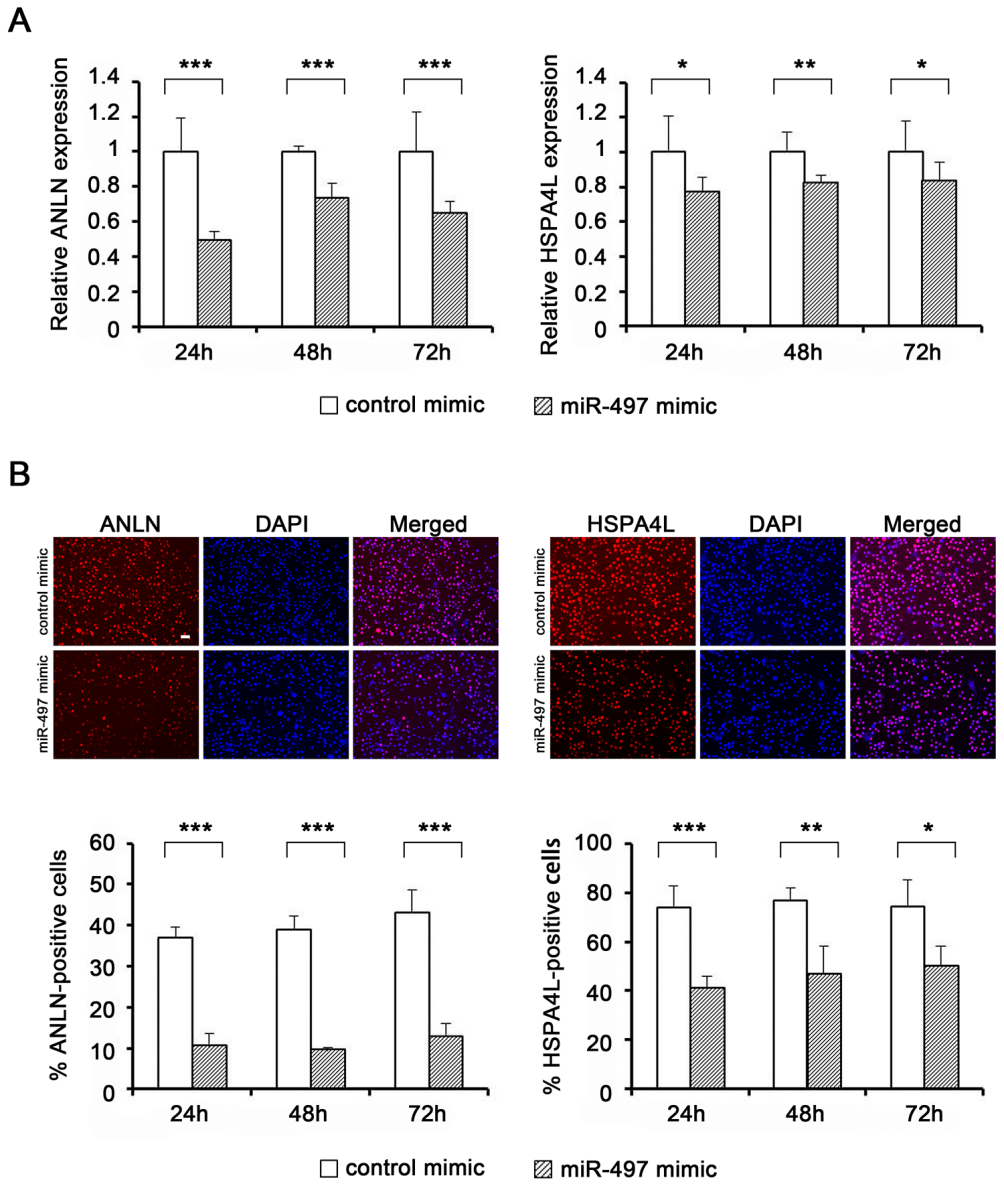
Target gene expression levels in NPC cells following transfection with miR-497 mimic **A.** Expression levels of *ANLN* (left) and *HSPA4L* (right) in HK1 cells (*n* = 4) after transfection with miR-497 mimic. Expression levels of target genes (*ANLN* and *HSPA4L*) were determined by quantitative RT-PCR and normalized against the corresponding levels of *GAPDH*. **B.** Protein expression levels of ANLN (left) and HSPA4L (right) in HK1/EBV cells, determined by ICC. Scale bar represents 50 μm. Numbers of positively staining cells and total cell number (determined by DAPI staining of nuclei) were analyzed using the ImageJ software, and the percentage of positively stained cells was calculated for each sample (*n* = 4) in three areas. *P*-values were calculated using the Student's *t*-test (*P < 0.05, **P < 0.01, ***P < 0.001).

Expression levels of miR-497 were significantly higher in NPC cells treated with miR-497 mimic for 72 h than in cells treated with the control mimic, although the levels of miR-497 gradually decreased after mimic transfection (Supplementary Fig. 2B–2D). Exogenous miR-497 significantly down-regulated the mRNA levels of *ANLN* (Fig. 5A, left) and *HSPA4L* (Fig. 5A, right), indicating that miR-497 can regulate the expression of these genes in NPC cells. Additionally, immunocytochemistry (ICC) analyses revealed that protein levels of ANLN (Fig. 5B, left) and HSPA4L (Fig. 5B, right) were significantly decreased by transfection with miR-497 mimic.

### Reduction of ANLN and HSPA4L potentially contributes to suppression of cell proliferation and migration, and induction of apoptosis, in NPC cells

**Figure 6:**
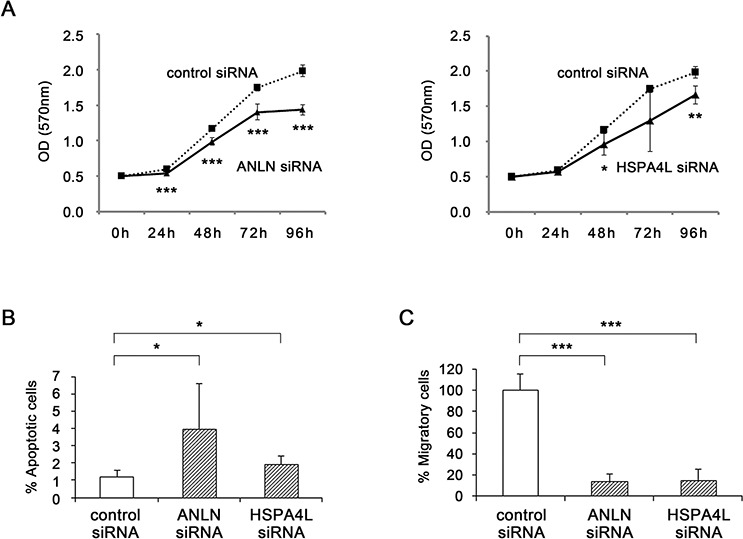
Functional analyses by silencing of ANLN and HSPA4L **A.** Cell proliferation was determined by MTT assay every 24 h for 96 h after transfection of CNE1 cells with *ANLN* siRNA (left) or *HSPA4L* siRNA (right) (*n* = 5). **B.** Apoptotic cells were detected by flow cytometry. CNE1 cells were transfected with negative control siRNA, *ANLN* siRNA or *HSPA4L* siRNA, for 72 h, and then stained with annexin V-FITC and PI and subjected to flow cytometry (*n* = 3). **C.** Cell migration assay in CNE1 cells transfected with negative control siRNA, *ANLN* siRNA, or *HSPA4L* siRNA. At 72 h after transfection, harvested cells were seeded in a migration chamber and cultured for 24 h. Migratory cells were stained, photographed under a microscope, and quantitatively analyzed (*n* = 3). Data are shown as means ± standard deviation. *P*-values were calculated using the Student's *t*-test (*P < 0.05, **P < 0.01, ***P < 0.001) compared with the control.

Quantitative RT-PCR confirmed that expression levels of *ANLN* and *HSPA4L* were significantly down-regulated in CNE1 cells after transfection of the corresponding siRNAs (relative quantities: *ANLN*, 0.51, *P* < 0.05; *HSPA4L*, 0.31, *P* < 0.05, respectively). To investigate the possible oncogenic function of *ANLN* and *HSPA4L*, we examined cell proliferation in siRNA-treated CNE1 cells for 96 h. Specific siRNA–transfected cells grew significantly more slowly than control siRNA–transfected cells (Fig. 6A).

The apoptosis rates in *ANLN* siRNA–transfected cells (4.02 ± 2.59%, *P* < 0.05) and *HSPA4L* siRNA–transfected cells (1.93 ± 0.50%, *P* < 0.05) were significantly higher than those in control siRNA–transfected cells (1.22 ± 0.34%, Fig. 6B).

Migratory cells were observed less frequently in *ANLN* and *HSPA4L* siRNA–transfected cells than control siRNA–transfected cells (Fig. 6C, *P* < 0.001 in both siRNAs). These results indicate that *ANLN* and *HSPA4L* induce cell growth, cell migration, and resistance to apoptosis in NPC cells.

## DISCUSSION

In this study, we analyzed the expression profiles of miRNAs in NPC using miRNA microarrays, and subsequently validated the findings by quantitative RT-PCR. Three EBV-encoded miR-BARTs and four hsa-miRNAs were significantly up-regulated, whereas six hsa-miRNAs were down-regulated, in NPC relative to NNE. According to a review by Hunt et al. [19], although technical replicates for microarrays typically show good reproducibility, there is a lack of interplatform agreement for expression profile data. Therefore, some of our results were coincident with previously reported microarray-based reports of dysregulation of miRNAs in NPC tissues, but we still obtained a different set of miRNAs. A recent review [20] indicated that several tumor-suppressor microRNAs, including miR-26a and miR-29c, are involved in NPC. In our preliminary study, which relied on previous reports, we measured these miRNAs by quantitative RT-PCR and observed significant down-regulation in NPC tissues relative to NNE tissues. Although miR-26a and miR-29c exhibited significant differences in our microarray analysis, the rank order of these miRNAs placed them outside of our candidate list. EBV is a critical etiological factor in NPC pathogenesis, and we found that three EBV-encoded miR-BARTs were highly expressed in all tissues from NPC patients. A threshold ΔCt value of 10 clearly distinguished between 18 NPC patients and 11 NNE patients. We also detected EBV-Encoded RNA (EBER) in NNE and NPC patients. All EBER-positive NPC cases had ΔCt values less than 10, and all EBER-negative NNE cases had ΔCt values greater than 10. Therefore, the nasopharyngeal tissues of all NPC patients in this study were infected with EBV, indicating that ebv-miR-BARTs, like EBER, could be used as biomarkers of infection in tissue. Despite the significant up-regulation of ebv-miR-BARTs in tumors, no ebv-miR-BARTs were significantly up-regulated in NPC plasma; this result was supported by a recent study that used RNA-Seq technology [21]. The recent surge of reports documenting circulating miRNAs in NPC patients supports their putative role as noninvasive cancer biomarkers. Among the dysregulated human miRNAs detected in tissues, miR-497 was concordantly down-regulated in plasma; therefore, miR-497 is a promising novel biomarker for diagnosis of NPC.

miR-497 is down-regulated in human tumors, such as prostate cancer [22], hepatocellular carcinoma [23], neuroblastoma [24], cervical cancer [25], breast cancer [26], colorectal cancer [27], and gastric cancer [28]. The mechanism responsible for miR-497 down-regulation remains unknown. Allelic loss is one reason for down-regulation of genes, but it occurs rarely in NPC [9]. On the other hand, EBV is an epigenetic regulator of host chromosomes. Because EBV-encoded miRNAs are highly elevated in epithelial carcinomas, they may target cellular genes important for host epigenetic programming, including DNA methylation [29]. Therefore, epigenetic silencing by DNA methylation may be one of the mechanisms underlying down-regulation of miR-497 in EBV-related NPC.

Several lines of evidence suggest that miRNAs play key roles in tumorigenesis, progression, invasion, or metastasis of NPC [30–32]. Although reduced expression of miR-497 was documented in a previous report [33], the biological function of miR-497 has not been characterized. Our functional studies using miR-497 mimic demonstrated that this miRNA efficiently suppresses cell proliferation and migration, and significantly induces apoptosis. Tumor growth *in vivo* was also suppressed by exogenous miR-497. Down-regulation of miR-497 may contribute to tumor growth and angiogenesis by targeting *HDGF* (hepatoma-derived growth factor) in non-small cell lung cancer [34]. *VEGFA* (vascular endothelial growth factor A) has also been predicted as a target gene [33], and we observed *VEGFA* up-regulation and neoangiogenesis in NPC in a recent study [35]. In addition, miR-497 plays a tumor-suppressive role in human cancer cell lines by targeting *BCL2*, thereby inducing apoptosis [36]. Zheng et al. [37] showed that IL-1–mediated *IL6* expression was significantly repressed by miR-497 via the MAPK/ERK pathway, suggesting that miR-497 may play a suppressive role in inflammation-related cancers such as NPC [38]. Notably, we demonstrated in this study that *ANLN* and *HSPA4L* are potential targets of miR-497. We confirmed the up-regulation of *ANLN* and *HSPA4L* in NPC primary tumors, and found that exogenous miR-497 indeed caused down-regulation of *ANLN* and *HSPA4L* in NPC cells. Consistent with this, RNA interference targeting *ANLN* and *HSPA4L* in NPC cells inhibited cell proliferation and migration and induced apoptosis. *ANLN*, a gene encoding the human homologue of anillin, an actin-binding protein of *Drosophila*, is essential for the organization of actin cables in the cleavage furrow, promotion of DNA synthesis, and activation of cellular motility. It also plays a key role in cytokinesis [14, 39, 40]. Up-regulation of *ANLN* in tumor specimens is a marker of poor prognosis in several cancers [14, 15, 41]. Our data are supported by a report that siRNAs against *ANLN* suppress growth, induce apoptosis, and inhibit migration in lung cancer cells [14]. By contrast, the heat shock protein (Hsp) 70 family facilitates cancer cell survival and growth in various human tumor cells by inhibiting apoptosis and promoting proliferation [42–45]. HSP70 has been suggested to play roles in the progression of malignant tumors [44]. *HSPA4L*, a member of the Hsp70 family, is highly expressed by leukemia cells and elicits humoral immune responses in leukemia patients [17]. Thus, on the basis of our results as well as recent reports, we suggest that down-regulation of miR-497 in NPC plays a causative role in NPC via up-regulation of *ANLN* and *HSPA4L*.

In conclusion, the concordant down-regulation of miR-497 in tissues and plasma make this miRNA potentially very useful as a diagnostic biomarker for NPC. This study demonstrated for the first time that miR-497 is a regulator of *ANLN* and *HSPA4L*. Down-regulation of this potent tumor suppressor may result in elevated expression of *ANLN* and *HSPA4L*, leading to NPC development and progression. This knowledge will facilitate development of novel targets for NPC therapy.

## MATERIALS AND METHODS

### NPC primary tumor biopsies and noncancerous nasopharyngeal epithelia (NNE)

This study was performed in accordance with ethical review committee approval notice (2009–07–07) from the First Affiliated Hospital of Guangxi Medical University, China, and ethical approval (No. 1116) from Mie University, Japan. In total, samples were taken from 18 patients (48.2 ± 11.2 years old, 12 males, 6 females) with NPC, and noncancerous nasopharyngeal epithelia (NNE) were obtained via tonsillectomy from 11 patients (46.9 ± 9 years old, 5 males, 6 females) with chronic nasopharyngitis, used as normal controls. All subjects were patients at the Department of Otolaryngology Head and Neck Surgery, First Affiliated Hospital of Guangxi Medical University, Nanning, China, and provided informed consent prior to participation. Diagnoses were made by experienced pathologists according to the World Health Organization (WHO) classification. The pathological diagnosis of all NPC samples was non-keratinizing carcinoma. Biopsy samples were stored in liquid nitrogen. Tissue and plasma samples were collected from the same patients.

### Extraction of RNA from tissues and miRNA microarray

Frozen tissues were treated with RNAlater ICE (Ambion) prior to RNA extraction. RNA containing miRNA was extracted using the mirVana™ miRNA Isolation Kit (Ambion, USA). One hundred nanograms of total RNA from seven NPC biopsies and five NNE samples were submitted to the Agilent miRNA microarray analysis service (Hokkaido System Science Co., Ltd., Sapporo, Japan). The array (SurePrint G3 Human miRNA, 8 × 60K, Rel. 16.0, Agilent Technologies, USA) contained more than 1300 probes for mature miRNAs, including EBV-related miRNAs.

### Detection of miRNA expression levels by quantitative RT-PCR

Tissue and cell samples were subjected to quantitative quantitative RT-PCR for miRNA. Reverse transcription for quantitative real-time PCR was performed using the miScript II RT kit (QIAGEN, Hilden, Germany); the miScript HiFlex buffer promotes conversion of all RNA species into cDNA, which can then be used in real-time PCR to quantitate mature miRNAs using miScript Primer Assays (QIAGEN). Quantitative real-time PCR was performed using the miScript SYBR Green PCR kit (QIAGEN). miRNA levels in tissues were normalized against the corresponding levels of the *RNU6B* snRNA. Relative expression levels of NPC relative to NNE were calculated using the ΔΔCt method.

### RNA extraction from plasma and measurement of miRNA expression levels by quantitative RT-PCR

For plasma collection, venous blood (5 ml) was collected using EDTA from noncancerous control and NPC patients before biopsy and before any therapeutic procedures, including radiotherapy. Blood samples were centrifuged at 1500 rpm for 15 min at 4°C to collect plasma and to remove residual cellular nucleic acids attached to cell debris. Plasma samples were stored at −80°C before miRNA extraction. The miRNeasy Serum/Plasma Kit (QIAGEN) was used to isolate small RNAs from 200 μl of plasma. A synthetic *C. elegans* miR-39 miRNA mimic (Syn-cel-miR-39, QIAGEN) and carrier RNA (0.94 μg, MS2 bacteriophage total RNA, Roche Applied Sciences, Indianapolis, IN, USA) were added to each plasma sample before RNA extraction. Isolated RNA was eluted with 15 μl RNase-free water.

The miScript II RT Kit (QIAGEN) was used to synthesize cDNA from 5 μl of the eluted RNA (containing miRNAs) in HiFlex buffer. Quantitative RT-PCR was performed using the miScript SYBR Green PCR kit (QIAGEN) and miScript Primer Assays. miRNA levels were normalized against the average of five reference miRNAs (miR-423-5p, miR-103a-3p, miR-191-5p, miR-425-5p, and miR-93), which were used as internal controls in plasma samples. Syn-cel-miR-39 was used to confirm RNA extraction efficacy. Relative quantities in NPC vs. NNE were calculated using the ΔΔCt method.

The five reference miRNA average mentioned above were ranked as the best miRNA standard by the online tool RefFinder (http://www.leonxie.com/) of the EST database. These five miRNAs exhibited no significant difference in plasma levels between NNE and NPC (Supplementary Fig. 1). In light of reports of up-regulation of miR-93 in NPC tissues [31, 32], we also measured miR-93 levels: miR-93 was up-regulated in NPC tissues (RQ = 2.78, *P* < 0.01) relative to NNE tissues, but plasma levels of miR-93 did not differ significantly between NPC and NNE patients, and there was no significant correlation between tissues and plasma (*r* = 0.109), suggesting that plasma miR-93 could be used as one of the normalization references.

### Cell culture

NPC cell lines (HK1/EBV, HK1) were a kind gift from Professor Tsao (Hong Kong University) [46–49]. The HK1/EBV cell line was established by stable infection of HK1 cells with recombinant EBV carrying the green fluorescent protein (EGFP) gene. NPC cell lines CNE1 and HONE1 were from Guangxi Medical University. Cells were maintained at 37°C in a 5% CO_2_ incubator. HK1/EBV and HK1 cells were cultured in RPMI1640 medium (Gibco, USA) supplemented with 10% fetal bovine serum (FBS, Gibco), 100 U/ml penicillin, and 0.1 mg/ml streptomycin. CNE1 and HONE1 cells were maintained in IMDM (Gibco) supplemented with 10% FBS and 0.1 mg/ml kanamycin.

### Transfection of miRNA mimic

Before transfection, NPC cell lines were maintained in serum-free medium. Cells were transfected with mirVana miRNA mimics (Ambion), or miRNA mimic negative control #1 (Ambion), at a final concentration of 30 nmol/L using GeneSilencer (Genlantis, San Diego, CA). After 4 h incubation, 1 volume of media containing 20% serum was added. The cells were then incubated at 37°C in a CO_2_ incubator for the indicated times, and used for subsequent experiments.

### Cell proliferation assay

NPC cells were seeded in 96-well plates at a density of 2,000 cells/100 μL/well and incubated at 37°C. Cell proliferation was assessed every 24 h for the indicated duration of time following transfection. Briefly, 10 μL of MTT (3-(4,5-dimethylthiazol-2-yl)-2,5-diphenyl tetrazolium bromide, 5.0 mg/mL; Sigma-Aldrich) was added to each well, and the plates were incubated for 4 h at 37°C. The culture media were removed, 100 μL DMSO was added, and the cells were incubated for 10 min on a shaker. Optical densities were determined at 570 nm on a Bio-Rad model 680 microplate reader, (Bio-Rad Laboratories, Hercules, CA, USA). OD values reflect the relative number of viable cells.

### ICC study

For ICC analysis, miRNA mimic–transfected cells were fixed with 1% (v/v) formaldehyde in phosphate-buffered saline (PBS) for 10 min at room temperature and washed three times with PBS. The cells were treated with 1% (v/v) Triton X-100 for 20 min, and then incubated with 5% (w/v) skim milk for 60 min at room temperature. Immunofluorescence was performed by incubation with mouse monoclonal anti-ANLN (1:100, Santa Cruz Biotechnology, Dallas, TX, USA), mouse monoclonal anti-HSPA4L (1:100, Santa Cruz Biotechnology), and rabbit polyclonal anti-caspase-3 (1:100, Santa Cruz Biotechnology) overnight at room temperature. The cells were then incubated with fluorescent secondary antibodies (Alexa Fluor 594–labeled goat anti-mouse IgG or Alexa Fluor 488-labeled goat anti-rabbit IgG; 1:400 each; Molecular Probes) for 2 h. Nuclei were stained with DAPI, and the stained cells were examined under a fluorescence microscope (BX53, Olympus, Tokyo, Japan). The number of positively-staining cells was analyzed using the ImageJ software (ver. 1.48).

### Apoptosis analysis by flow cytometry

To measure apoptosis, expression of Annexin V-FITC and exclusion of propidium iodide (PI) (TACS Annexin V-FITC, Trevigen, Inc., Gaithersburg, MD) were detected by double-label flow cytometry. Seventy-two hours after transfection, cells were collected, washed once with PBS, and washed again with 400 μL binding buffer. Samples were incubated with 100 μl Annexin V-FITC reagent in the dark for 15 min at room temperature, and then the volume was adjusted to 500 μl with binding buffer. Fluorescence was measured on a flow cytometer (BD Biosciences, FACSCanto II, San Jose, CA, USA) within 1 h for maximal signal.

### Cell migration assay

Cell migration assays were performed using the CytoSelect Cell Migration Assay Kit (8 μm pore size of membrane filter; Colorimetric Format; Cell BioLabs, Inc., San Diego, CA, USA). Briefly, 72 h after transfection, NPC cell suspension (0.15 × 10^6^ transfected cells/well) in serum-free medium was placed in the upper chamber, and media containing 10% FBS was placed in the lower well of the migration plate. After incubation for 24 h and removal of non-migratory cells, cells that migrated through the filter were stained and photographed under a microscope (100 × and 200 × magnification) for each filter in three areas. Migratory cells were counted, and the average number of migratory cells in the control condition was assigned as 100%.

### *In vivo* tumor growth assay

Five-week-old male BALB/c athymic nu/nu mice (Japan SLC, Inc., Hamamatsu, Japan, weight range: 16–19 g) used for these experiments were maintained at the Institute of Laboratory Animals at Mie University. All animal experiments were performed according to the Mie University guidelines for laboratory animals (approval No. 26–19). Animals were housed in ventilated caging conditions under a 12-h dark/light cycle at constant humidity and temperature. Animals were permitted free access to sterile water and standard laboratory chow. Subcutaneous xenografts were established by inoculating 2 × 10^6^ miR-497 mimic–transfected cells into the left flank, or an equal number of control mimic–transfected cells into the right flank. The mice were observed for tumor formation, and tumor volume was measured with a caliper (model 530–312; range 0–150 mm; Mitutoyo, Kawasaki, Japan) and calculated using the formula: *V*=*L* × *W* × *H* × π/6, where L, W, and H represent tumor diameters in three mutually perpendicular planes. After the mice were sacrificed by cervical dislocation, the tumors were recovered and the wet weights of each tumor were determined.

### Detection of gene expression using mRNA microarray and quantitative RT-PCR

Fifty nanograms of RNA from seven NPC biopsies and five NNE samples were subjected to Agilent SurePrint G3 Human GE microarray analysis (Hokkaido System Science Co., Ltd.). The array (SurePrint G3 Human GE 8 × 60K, 1 color) contained probes for more than 20,000 genes for gene expression analysis. Probes with significant signal in samples were used for data analyses (22,922 probes).

SYBR Green RT-qPCR assays were used to assess mRNA expression in tissue and cell samples. The miScript II RT Kit (QIAGEN) was used to synthesize cDNA in HiFlex buffer. Quantitative PCR was performed using the miScript SYBR Green PCR kit and QuantiTect Primer Assays (QIAGEN). mRNA levels were normalized against the corresponding levels of *GAPDH* mRNA, and relative quantities (RQ) of NPC vs. NNE were calculated using the ΔΔCt method.

### IHC study

For IHC analysis, standard immunoperoxidase methods were used to examine the distribution of ANLN and HSPA4L in NPC tissues and normal controls. After deparaffinization and rehydration, antigen was retrieved in 5% urea buffer by microwave heating for 5 min, and then incubated in 1% H_2_O_2_ for 30 min to block endogenous peroxidase activity. Sections of 3-μm thickness were incubated overnight at room temperature with the following antibodies: mouse monoclonal anti-ANLN (1:100, Santa Cruz Biotechnology) and mouse monoclonal anti-HSPA4L (1:100, Santa Cruz Biotechnology). For mouse antibodies, the sections were incubated with biotinylated anti-mouse IgG for 3 h, and then incubated with avidin–biotin complex (Vectastain ABC kit, Vector Laboratories, Burlingame, CA, USA) for 2 h. Sections were then incubated with 3,3′-diaminobenzidine (DAB substrate kit; Vector Laboratories). Nuclei were counterstained with hematoxylin.

IHC grading based on intensity and frequency of staining was performed by two independent investigators without knowledge of the patients’ clinicopathological features. Staining intensity was scored as negative (0), weak (+1), moderate (+2), or strong (+3). Frequency of positive cells in specific areas was scored as negative (0), less than 25% (+1), 25–50% (+2), 51–75% (+3), or more than 75% (+4). IHC grading was assigned an IHC score as follows: −, negative expression (0); +, weak expression (1 − 3), ++, moderate expression (4 − 6); +++, high expression (7 − 9) or ++++, very high expression (10 − 12).

### Candidate target gene siRNA transfection

Small interfering RNA (siRNA) for candidate target genes *ANLN* (SDO-1006-1005899, GENE_ID54443) and *HSPA4L* (SDO-1006-1071379, GENE_ID22824), as well as negative control siRNA (SN-1001), were synthesized by BIONEER (Daejeon, South Korea). The transfection was performed using GeneSilencer. Briefly, CNE1 cells were seeded at 1 × 10^5^/well in 6-well plates 1 day prior to transfection with 1000 ng of siRNA. The cells were collected after 24 h, and the expression levels of target genes were confirmed by real-time PCR in CNE1 comparing specific siRNA-treated and negative control siRNA–treated cells.

### Statistical analysis

Statistical analyses were performed using the SPSS19 software package. Data are presented as means ± standard deviation. Student's *t*-test was used to compare differences in ΔCt values between two groups. Statistical differences of IHC grading and score were determined by the chi-square test and Mann–Whitney U test, respectively. A *P*-value less than 0.05 was considered to be statistically significant.

## SUPPLEMENTARY MATERIALS FIGURES AND TABLE



## Abbreviations

miRNA: microRNA
NPC: nasopharyngeal carcinoma
NNE: noncancerous nasopharyngeal epithelia
EBV: Epstein-Barr virus
BARTs: BamHI A Rightward Transcripts
siRNA: small interfering RNA
ANLN: anillin, actin-binding protein
HSPA4L: heat shock 70 kDa protein 4–like
IHC: immunohistochemistry
ICC: immunocytochemistry
