# Supplementary material for: The potent tumor suppressor miR-497 inhibits cancer phenotypes in nasopharyngeal carcinoma by targeting ANLN and HSPA4L
Source: Oncotarget. 2015 Oct 14;6(34):35893–907. doi: 10.18632/oncotarget.5651 (PMC4742149; doi:10.18632/oncotarget.5651)
Supplement: Supplementary file 1 [file oncotarget-06-35893-s001.pdf]

SUPPLEMENTARY FIGURES AND TABLE

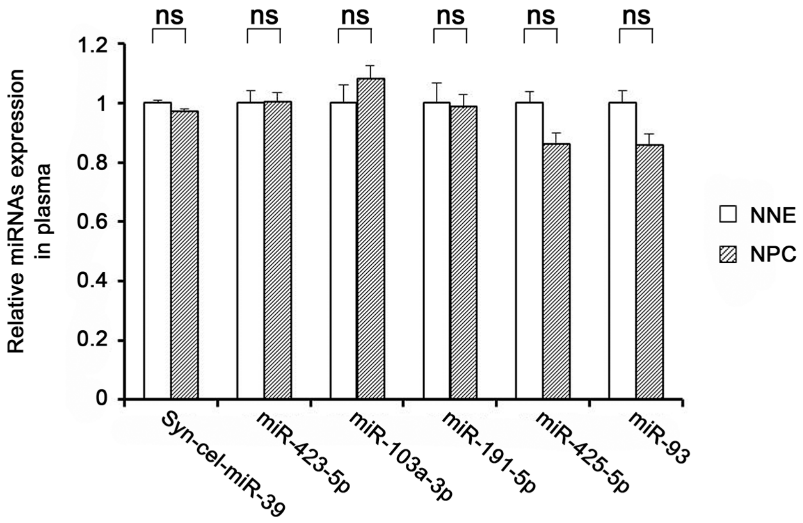

Supplementary Figure S1: MiRNAs expression levels in plasma. Expression levels of spike-in syn-cel-miR-39 (cel-39) and 5 reference hsa-miRNAs (miR-423-5p, 103a-3p, 191-5p, 425-5p and 93) in plasma with no normalization. *P*-values were calculated using the Student's *t*-test (ns, no significant difference). The relative quantity of NNE was set at 1.

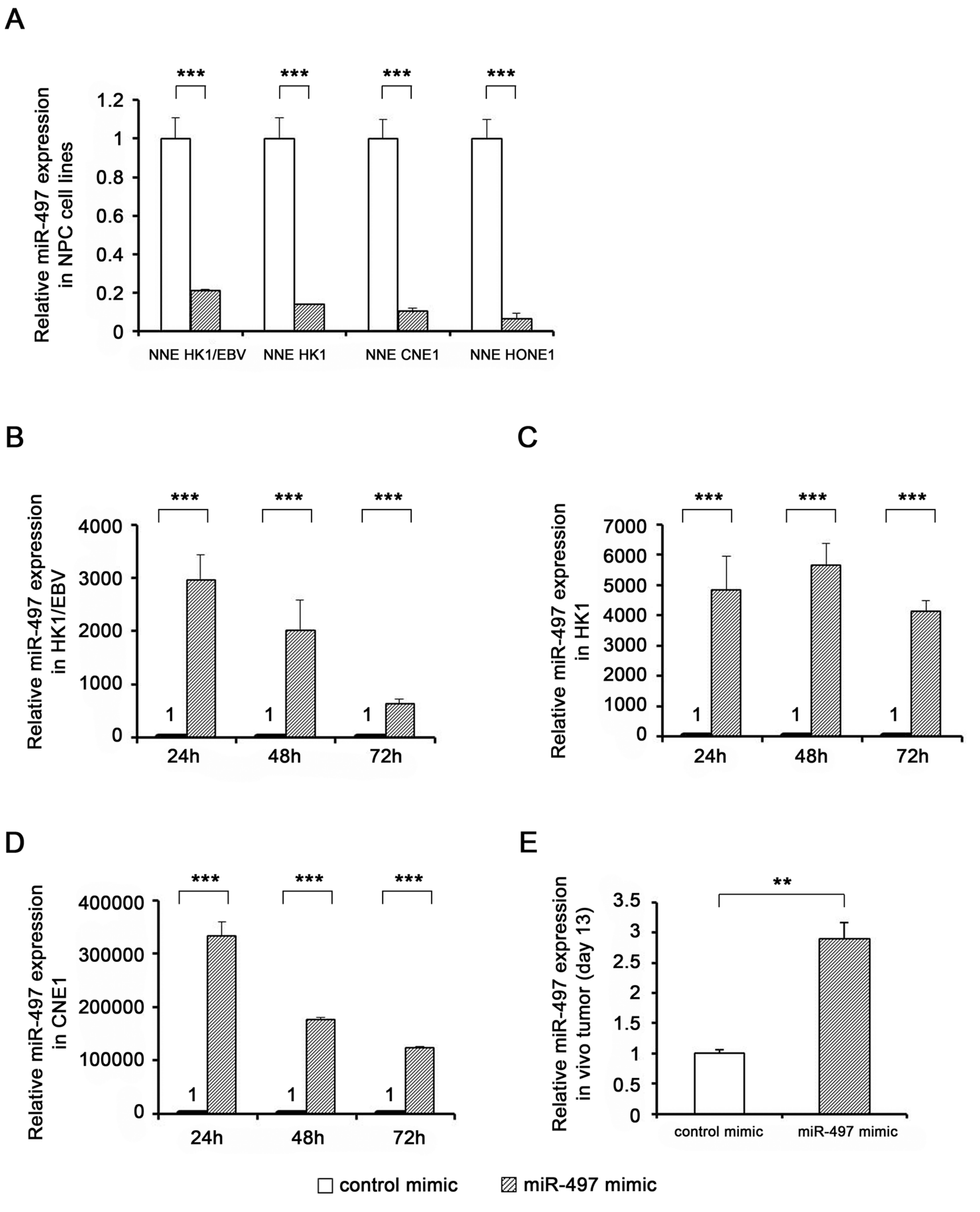

Supplementary Figure S2: MiR-497 expression levels in NPC cells by the transfection of miR-497 mimic. A. Expression levels of miR-497 in NPC cells were compared with NNE tissues ($n = 11$). After transfection, the relative expression levels in (**B)** HK1/EBV, (**C**) HK1 and (**D**) CNE1 ($n = 4$) at the indicated time points, and (**E**) in tumor xenograft *in vivo* of transfected-HONE1 cells ($n = 7$) at day 13 were compared between miR-497 mimic and control mimic-transfected conditions. Expression levels were determined by quantitative RT-PCR and normalized to *RNU6B*. *P*-values were calculated using the Student's *t*-test (**$P < 0.01$, ***$P < 0.001$).

Supplementary Table S1: MiRNA relative expression in tissues and plasma analyzed by qRT-PCR

miRNA name	&Fold change in tissue	&Fold change in plasma	$Correlation efficiency (*r*)	$*P*-value
**(A) Epstein-Barr virus-related miRNAs**				
ebv-miR-BART22	14018.28***	3.76	0.229	0.233
ebv-miR-BART1-3p	10418.09***	1.61	0.113	0.560
ebv-miR-BART9	6657.04***	1.72	0.190	0.323
**(B) Human miRNAs**				
**1) Up-regulated miRNAs**				
hsa-miR-205	10.55***	1.37	0.189	0.326
hsa-miR-182	5.68***	1.13	0.044	0.736
hsa-miR-135b	6.16***	0.99	−0.041	0.832
hsa-miR-455-3p	2.82***	1.07	−0.208	0.287
**2) Down-regulated miRNAs**				
hsa-miR-145	0.21**	0.98	0.247	0.196
hsa-miR-497	0.39***	0.36**	0.490	0.007##
hsa-miR-150	0.25***	1.02	0.079	0.682
hsa-miR-195	0.90	–	–	–
hsa-miR-342-5p	0.31***	1.35	0.013	0.946
hsa-miR-143	0.56	–	–	–
hsa-miR-34b*	0.17**	0.95	−0.008	0.969
hsa-miR-100	0.26***	0.93	0.123	0.525

&: The relative quantity of NNE was set at 1. -: not detected.
*: $P < 0.05$,
**: $P < 0.01$,
***: $P < 0.001$ compared to NNE by Student *t*-test.
$: Pearson's correlation efficiency and *P*-value between tissues and plasma (##: $P < 0.01$).
